# Wrapping Our Heads around Endocytosis

**DOI:** 10.1371/journal.pbio.1000207

**Published:** 2009-09-29

**Authors:** Caitlin Sedwick

**Affiliations:** Freelance Science Writer, Encinitas, California, United States of America

**Figure pbio-1000207-g001:**
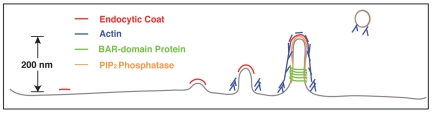
The progression of vesicle formation in yeast.

[Fig pbio-1000207-g001]Eukaryotic cells use a process called endocytosis to take up materials from their environment and package them for use within the cell. The best-studied form of endocytosis—receptor-mediated endocytosis—has several stages. In the first step, the cell's outer membrane dimples to form a pocket. A protein collar then assembles around this pocket, forming a thin neck leading to a bulb of membrane. Next, this bulb pinches off to form a membrane-bound sphere, or vesicle.

Yeast and mammalian cells follow the same general steps of endocytosis (forming the pocket, collaring it, and pinching it off) and use similar ensembles of proteins to achieve them. Both cells use a protein called clathrin to coat the sites where pockets form, and pocket formation is associated with a burst of actin assembly. Mammalian cells also rely on the enzyme dynamin to collar and help pinch off vesicles. Yeast, meanwhile, manage to pinch off vesicles efficiently without dynamin, raising questions about the fundamental pinching mechanism. Yeast and mammals also use Bin-Amphiphysin-Rvs (BAR)–domain proteins to generate and sense membrane curvature.

How do mammalian and yeast cells coordinate the activities of these various participants to form endocytic vesicles? In this issue of *PLoS Biology*, Jian Liu and colleagues theorize that endocytosis is coordinated in both types of cells by processes that affect and respond to the curvature of cell membranes.

In both mammalian and yeast cells, the membrane lipid phosphatidylinositol (4,5) bisphosphate (PIP2) plays a crucial role in endocytosis. Membrane levels of PIP2 are regulated by enzymes that phosphorylate (that is, add a phosphate group to) lipid precursors to create more PIP2, and enzymes called phosphatases that dephosphorylate (remove a phosphate group from) PIP2 to reduce its levels. Early on in endocytosis, there are high levels of PIP2 in the membrane that serve to recruit clathrin or other coat proteins to the site where membrane dimples will form. Later, though, PIP2 levels must be reduced to allow these coating proteins to drop off and be replaced by a different set of proteins that will direct trafficking of the vesicle within the cell.

Liu and colleagues wondered whether this cycle regulating PIP2 levels might also be responsible for other critical steps in endocytosis, such as the pinching off of vesicles. They created a conceptual and computational model to help explore this idea. In their model, the coat proteins that are recruited by PIP2 to the endocytic site cause the cell membrane to deform into a dimple. Collar proteins bind better to curved membranes, so the formation of this initial curve promotes the recruitment of collaring proteins. Collar proteins also increase membrane curvature where they attach, so their recruitment is a self-reinforcing process.

As the growing protein collar constricts the neck of the pocket, the membrane at the end of the collar bulges out into a bulb. The lipid membrane in this bulb is stretched into a sharp curve. Recent work by other groups has demonstrated that some lipid phosphatases are more efficient at cleaving PIP2 as membrane curvature increases. Therefore, Liu and coauthors suggest that PIP2 in the bulb, and especially at the interface between the bulb and the neck of the pocket, gets quickly dephosphorylated as the neck gets more tightly curved.

Nothing exciting would happen if PIP2 levels decreased universally across the surface of the membrane pocket. But, what if PIP2 in the neck region is protected from dephosphorylation by the collar proteins, so that only PIP2 on the bulb is vulnerable? The dephosphorylated lipid takes up less space in a membrane, so differences in PIP2 levels at the neck/bulb juncture would create a circumferential force that acts like a noose. This noose would collapse the membrane at the neck/bulb interface, pinching off the vesicle.

The authors' model encompasses these ideas, and computer simulations using the model recapitulate what is observed in nature. The model successfully describes the general process of endocytosis and, what's more, allows the authors to make specific predictions about what may change if certain protein players are tinkered with experimentally (e.g., through mutation). Although these predictions have yet to be explicitly tested, the model offers a roadmap to finally understanding the cascade of events involved in endocytosis.

**Liu J, Sun Y, Drubin DG, Oster GF (2009) The Mechanochemistry of Endocytosis. doi:10.1371/journal.pbio.1000204**

